# ARDS Studies in Critical Care Journals: How Representative Are the Patients Studied?

**DOI:** 10.1155/ccrp/4060643

**Published:** 2025-09-10

**Authors:** Jennifer Varallo, Tarek Nahle, Peter Galiano, Ricardo Jaime Orozco, Christopher Ambrogi, Adam Green, Jean-Sebastien Rachoin

**Affiliations:** ^1^Department of Medicine, Cooper University Health Care, Camden, New Jersey, USA; ^2^Department of Medicine, Cardio-Oncology Program, Medical College of Georgia at Augusta University, Augusta, Georgia, USA; ^3^Department of Medicine, Inspira Health, Vineland, New Jersey, USA; ^4^Cooper Medical School of Rowan University, Camden, New Jersey, USA

**Keywords:** acute respiratory distress syndrome, ARDS, inclusion, intensive care, reporting

## Abstract

**Purpose:** Implicit bias in medicine is widespread, with minority populations historically underrepresented in research. Studies have shown racial and ethnic disparities in patient outcomes, including in acute respiratory distress syndrome (ARDS). This study examines the representation of minority patients in ARDS research in the USA.

**Methods:** We examined the 1000 most cited ARDS studies from 2011 to 2021 in the top five critical care journals: AJRC, CHEST, Critical Care, CCM, and ICM.

**Results:** 211 met the inclusion criteria, with 90 providing racial and ethnic demographic information for analysis. These included 17 in AJRC, 36 in CCM, 18 in CHEST, 11 in CC, and 8 in ICM. The average number of citations was 53 (±63). Publications peaked from 2015 to 2017 (15/year), while 2021 had the fewest. The mean patient count was 15,168, including 42 prospective, 29 randomized controlled, and 19 retrospective studies. Eighty-eight studies reported an average patient age of 53 years (±6), and 72% (±15%) of patients were White. Thirty-five studies reported only White patient demographics, while 53 included Black patients, 29 discussed Hispanic patients, 21 mentioned Asian patients. Most studies reported an average of 43% female participants, with no correlations found regarding White patient numbers, publication year, citations, or journals.

**Conclusion:** A substantial number of highly cited studies about ARDS published in prominent critical care journals did not have detailed information regarding the racial composition of the patient population, and a large majority included overwhelmingly White patients and a preponderance of male gender patients.

## 1. Introduction

Acute respiratory distress syndrome (ARDS), a common type of severe respiratory failure, demands urgent attention and remains a persistent challenge despite decades of research and clinical innovation. This condition continues to be a major contributor to morbidity and mortality in the intensive care setting. Despite advancements in clinical research, ARDS mortality rates remain elevated, being around 45% in-hospital, 38% in the intensive care unit (ICU) setting, and 32% for 60 day mortality [1]. This persistent burden underscores the multifactorial nature of ARDS prognosis.

Various physiological factors, including the severity of lung injury, comorbid conditions, and ventilatory parameters, influence clinical outcomes. However, emerging evidence suggests that demographic and social factors such as race, sex, age, and body mass index (BMI) also play a critical role in shaping patient clinical trajectories [2–5]. For example, a 2019 retrospective study found females with severe ARDS had a higher mortality risk than their male counterparts [6]. Another retrospective study found a higher risk of mortality in Black and Hispanic patients when compared to White patients with ARDS [7]. These sociodemographic factors may impact individual risk and access to care, highlighting the importance of considering the broader context in which ARDS occurs and progresses. Despite the demonstration of poor outcomes in these vulnerable populations, minority groups are historically underrepresented in medical research.

Many studies have shown that minority populations remain underrepresented in clinical research in the United States despite the US's diversity, despite the Black population consisting of 41.1 million individuals (12.4%) in the United States in 2020, compared to 38.9 million (12.6%) in 2010, growing in absolute numbers but decreasing in percentage of the overall United States population [8–11]. This disparity is seen across general medicine and medical subspecialties, including critical care. A recent epidemiologic meta-analysis found Black patients were significantly underrepresented in critical care randomized controlled trials (RCTs), limiting the generalizability of studies to the public [12]. Leading critical care societies, such as the American Thoracic Society (ATS), have recognized minority representation as crucial to advancing critical care research and formally called for increased inclusion when recruiting for studies [13].

As ARDS has been shown to affect minority populations disproportionately, it becomes increasingly important to recruit diverse study populations representative of the general public. Considering this, we performed this study to examine the representativeness of ARDS investigations across leading high-impact critical care journals to better characterize the identification and prevalence of minority populations in ARDS research.

## 2. Materials and Methods

### 2.1. Journal and Article Selection

The Web of Science (WoS) Core Collection, provided by Clarivate Analytics, was chosen due to its comprehensive data coverage and widespread use in bibliometric research. We conducted a descriptive analysis focusing on the most cited articles related to ARDS conducted between 2011 and 2021 in the most prominent five journals in critical care that were selected based on their impact factor rankings as provided by Journal Citation Reports. These journals were the American Journal of Respiratory and Critical Care Medicine (AJRC), CHEST, Critical Care (CC), Critical Care Medicine (CCM), and Intensive Care Medicine (ICM). Using citation metrics, we identified the top 1000 most-cited ARDS articles from these high-impact journals. Search terms regarding ARDS were chosen after a meticulous examination of published literature in that domain and based on frequently used keywords. The search strategy included the terms: “ARDS” (Topic) OR “Acute Respiratory Distress Syndrome” (Topic) AND “2011–2021” (Year Published) AND “article” (Document Type). Two authors (JV, PG) conducted screening and data collection of pertinent information necessary for subsequent analysis. Relevant details included the year of publication, title, authors, affiliated institutions, publishing journal, abstracts, references, citations, and the impact factor.

Bibliometric data were retrieved from the WoS database and exported into Excel for detailed analysis. Any discrepancies identified were cross-checked and validated against the original WoS records.

### 2.2. Inclusion and Exclusion Criteria

Among the top 1000 cited articles, we included in our study those that met the following criteria: focused on adult patients aged 18 years or older; included at least one patient cohort from the United States; and provided demographic information within their analyses, specifically data on race, ethnicity, sex, or socioeconomic indicators. Studies designed as reviews, those not including human participants, targeting pediatric populations, those that did not include a patient cohort from the United States, did not provide the necessary demographic data, or were not published in English were excluded from the study. After the initial screening, four different authors (JV, PG, RJO, and CA) conducted an in-depth analysis of the articles to check if they met our inclusion criteria.

### 2.3. Study Endpoints

In this study, the primary endpoint was the proportion and level of detail of racial and ethnic demographic reporting in the most-cited ARDS studies published in high-impact critical care journals that included at least one U.S. patient cohort. Secondary endpoints included the assessment of representation levels of female patients within these studies and the evaluation of temporal trends in demographic reporting and representation over the study period.

### 2.4. Data Extraction

We systematically extracted detailed demographic information from each included article, specifically race and ethnicity (Asian, Black, Native American/Alaska Native, Hispanic, White, Multiracial, Other), sex, average age, average BMI, and study characteristics such as the type of the study (RCT, prospective observational, or retrospective observational), total number of patients enrolled, and study duration (start and end years).

### 2.5. Data Synthesis and Analysis

Descriptive statistics were used to summarize the collected data. Continuous variables were expressed as means with standard deviations, and categorical variables were expressed as percentages. Trends in demographic representation and methodological characteristics were assessed to examine the generalizability and inclusivity of patient populations in high-impact ARDS research. Differences in categorical variables are assessed using the chi-square test and continuous variables using the one-way ANOVA test. All analyses were conducted using SPSS version 26 (IBM Corporation, Armonk, NY), with *p* < 0.05 considered statistically significant.

## 3. Results

### 3.1. Research Output

**Figure 1:**
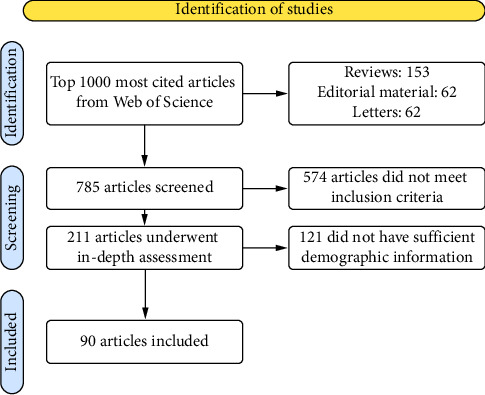
Article selection process.

After the initial screening of the 1000 most cited ARDS articles, 211 studies met the inclusion criteria. Following a full-text review of the presence of racial and ethnic demographic data, 90 of the initial 211 studies were found to contain demographic information including race and ethnicity for inclusion in the analysis. 78 studies of the original 211 included solely information related to age and sex, 32 included age and sex and BMI, and 10 others which were excluded. The article selection process is summarized in Figure 1.

These studies were distributed across journals as follows: CCM (*n* = 36), CHEST (*n* = 18), AJRC (*n* = 17), CC (*n* = 11), and ICM (*n* = 8). The average number of citations per study was 53 (±63). Study designs included 42 prospective studies, 29 RCTs, and 19 retrospective cohorts. A list of the articles included is detailed in Supporting Table 1. The included studies encompassed a wide variety of patients, with 78 studies including patients intubated/mechanically ventilated, 5 studies in patients with post-intubation status, 3 studies in patients at-risk of intubation status, and 2 studies in patients intubated/weaning.

**Figure 2:**
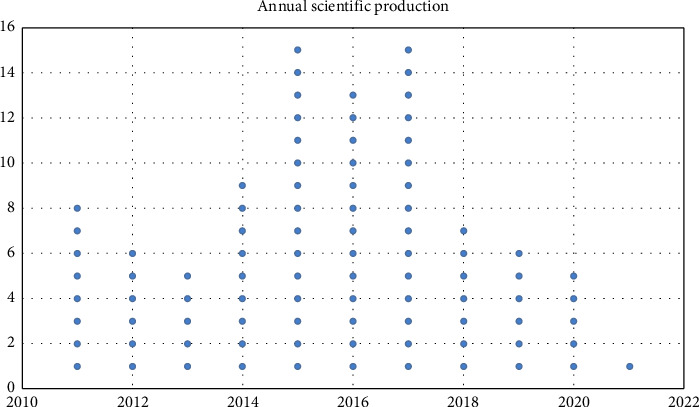
Trends in annual research output in ARDS (2011–2021), which include an adult US cohort and patient demographic data.

The publication of articles that met inclusion criteria peaked between 2015 and 2017, with 15 studies published in both 2015 and 2017 and 13 studies published in 2016, while only one eligible study was published in 2021. The surge in publications started after 2013, and then the numbers decreased after 2017. Annual publication volume trends are displayed in Figure 2.

### 3.2. Demographic Reporting

The total number of patients included was 1,365,147 with a mean of 15,168 (±123,161). Age was reported in 88 studies, with a mean of 53 years (±6); gender in 89 studies, with a median female representation of 43% (range: 15.8%–67%); and BMI figured in 28 studies, with an average of 29 (±5) kg/m^2^.

**Figure 3:**
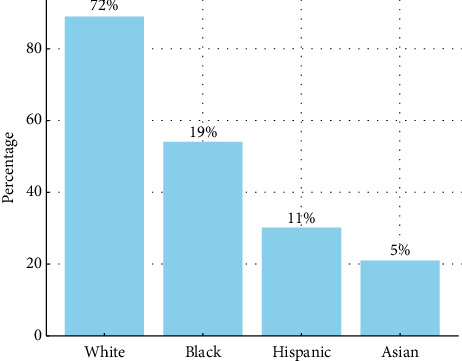
Percentage of racial and ethnic groups data in ARDS studies reporting demographic data.

All but one study reported the number or proportion of White patients, averaging 72% (±15%). However, 35 studies reported only White patient data without reference to other racial or ethnic groups. Black patients were reported in 53 studies, Hispanic patients in 29 studies, and Asian patients in 21 studies. Native American/Alaska Native representation was noted in 8 studies and multiracial patients in 3. Additionally, 42 studies included an unspecified “other” category. The average number of patients identifying as Black who were included was 19% (range 0.3%–62.3%), for Hispanic patients was 11% (range 1.2%–31.5%), and for Asian patients was 5%. A diagram of racial representation is presented in Figure 3.

Out of the 53 studies reporting Black patient proportions, 37 were based in the United States, with the leading states being Tennessee, Massachusetts, California, and Pennsylvania; 5 were multicentered studies between the United States and Canada; and 11 were network studies across multiple countries.

### 3.3. Demographic Data Based on Publication Journal

In Table 1, we present demographic data based on the publication journal. Most studies were published in CCM (36), followed by CHEST (18), AJRCC [14], CC (11) and ICM [8]. The average number of citations was highest in AJRCC (105.2) and lowest in CC (31.8). The lowest number of patients enrolled was 291.8 (CC), and the highest was 62,625 (CHEST). Age of patients was relatively similar in all studies, as well as the proportion of females. The proportion of the White race ranged from 69.9% to 72.7% and that of Black patients from 13.2% to 21.6%.

## 4. Associations

In Table 2, we present data for studies that reported only White race and those who reported multiple races. Studies that reported only White race were published in AJRCC (4, 23.5%), CC (3, 27.3%), CCM (16 (44.4%), CHEST (6, 33.3%), ICM (6, 75%). There were 20 (47.6%) prospective cohorts, 5 (26.5%) retrospective studies, and 10 (34.5%) RCTs. Studies with only the White race has an average citation of 46.7 vs 57.7 for studies that reported all races. No consistent associations were found between studies that reported only White patients and their publication year, citation count, or journal of origin.

## 5. Discussion

This study assessed the representativeness of patients in ARDS studies in the United States. We found that a large proportion of the most cited articles in the most impactful journals dedicated to critical care do not describe the racial composition of participants. Additionally, of those who did, non-Hispanic White patients were predominantly represented. Of the studies that reported demographic information, the reporting was also inconsistent, with some characterizing patients as “White” or “non-White” versus others listing specific racial and ethnic groups. Additionally, the fact that out of the original 211 studies, only 90 included age, sex, and racial and ethnicity data underscores a primary finding of our investigation. Missingness of racial or ethnic composition of participants is a critical data gap in itself, reinforcing our conclusion that the generalizability of findings from highly impactful critical care literature is often limited by a lack of fundamental demographic reporting. Furthermore, there were a larger number of male patients than female patients in these studies. These findings demonstrate the difficulty in generalizing the findings to the public, as their demographic information was unknown or not representative. Ensuring representative enrollment and consistent demographic reporting would be essential in developing inclusive and effective interventions across all population subgroups.

The proportion of Black patients reported across studies varied widely, ranging from as low as 0.3% to as high as 62.3%, with a mean representation of 19%. While this may initially suggest adequate Black patient inclusion, the wide range reflects a substantial inconsistency in reporting and sampling practices across studies, as seen by the heterogeneity in reporting of race and ethnicity across the studies between years, especially with the relatively stable percentage of the Black population between 2010 and 2020. This divergence raises concerns about potential enrollment biases, lack of standardized reporting, or structural barriers to inclusion, especially since several studies from the same country/state report different percentages of ethnicities, with most of them being inconsistent with the geographical demographic characteristics. Moreover, without consistent stratification or subgroup analysis by race, the utility of such inclusion for informing equitable clinical decision-making remains limited. Given known disparities in ARDS incidence, outcomes, and access to care among Black populations, uniform and detailed reporting is essential to understand how findings may differentially apply across racial and ethnic groups.

Like the variable identification and reporting of minority groups, patient outcomes in underrepresented populations vary amongst ARDS literature [15]. While some studies have associated certain racial and ethnic groups with poor outcomes in ARDS, others have shown a protective effect. For example, some studies showed that the Black race seemed to have a protective effect on ARDS incidence [16]; others noted an increased risk in this population [17]. Differences in demographic reporting may have influenced the discrepancy in the findings of these studies. Additionally, the process of racial identification, such as self-reported identification, may have impacted the representation of certain racial or ethnic groups, especially if a participant considered themselves more “racially ambiguous” than the provided responses.

Though unknown, the cause of disproportionate outcomes in minority populations affected by ARDS is likely multifactorial and includes socioeconomic, environmental, and biochemical factors. Certain genetic polymorphisms found in particular racial and ethnic groups have been found to be associated with worse clinical outcomes. For example, it was shown that Black patients who have the *rs2814778* polymorphism in the gene encoding for the Duffy antigen/receptor for chemokines (DARC) were more susceptible to worse clinical outcomes, theorized to be a consequence of increased IL-8 levels [14]. Additionally, Black patients are at higher risk of enduring violence-induced TBI, a known risk factor for the development of ARDS [15, 18]. The interplay of sociodemographic and biologic factors on ARDS outcomes in minority populations is not entirely understood, partially due to the lack of representation of vulnerable populations within ARDS research. Our findings emphasize the need for more systematic and standardized approaches to participant sampling to bring reliable evidence to present practice.

In a study conducted by Papoutsi et al., the representation of minorities in clinical trials was found to be 30.4% (95% CI 27.7%–33.2%) with a range of 24.9%–38.5%, which is much narrower than the ranges in our study [19]. This may be explained by the stricter nature of recruitment in RCTs compared to other types of studies. However, the broader discrepancies seen in different study designs may not be solely attributed to differences in enrollment practices. Additional contributing factors, such as underreporting of demographic variables as demonstrated in this study, a lower degree of follow-up in minorities, and systemic disparities in access to care, likely influence the observed variation in minority representation [20, 21].

Strengths of this study included a focus on high-impact literature, ensuring that our findings reflected research with the greatest influence on guideline development and clinical practice. Comprehensive and systematic screening with clear inclusion criteria ensures the optimal reduction in selection bias and improved methodological rigor. Data extraction from full-text articles ensures the accuracy and completeness of information on selected studies and populations. Additionally, this study is generalizable to the United States population, as it includes exclusively studies that have at least one cohort from the United States. Finally, all studies included are relatively recent, which prevented the results from being skewed by outdated patient selection and enrollment practices.

Conversely, this study has several limitations. Many included studies are retrospective, with patient inclusion limited by the scope and structure of available datasets. Additionally, our findings heavily depend on reporting practices; numerous studies did not include race or ethnicity in their baseline characteristics, which may lead to underestimating minority representation due to underreporting. Moreover, participant enrollment in clinical trials may reflect the geographic distribution of study centers, which can influence the racial and gender composition of the recruited cohorts and contribute to the observed variability in demographic representation [22]. Furthermore, selection bias may be present since most studies selected are open access, which induces wider access for researchers and could potentially result in a higher citation count. Additionally, although our study included studies from the five highest impact factor journals in critical care, articles with higher citation count could be published in different journals. Finally, a higher citation counts might not imply higher study impact and clinical relevance.

## 6. Conclusion

Our analysis demonstrates the underrepresentation and underreporting of racial compositions in many of the most influential ARDS studies published in high-impact critical care journals. Among reporting studies, there was an overrepresentation of White individuals and predominance of male participants. This pattern raises concerns about the inclusivity, representativeness, and generalizability of ARDS literature and emphasizes the importance of improving demographic transparency in future research.

## Figures and Tables

**Table 1:** Demographic data based on the publication journal.

Journal	CCM	Chest	AJRCC	CC	ICM	All
Number of manuscripts	36	18	17	11	8	90
Number citations	37.8 (±20.1)	51.8 (±45.2)	105.2 (±124.7)	31.8 (±15.7)	46.1 (±23.9)	53.3 (±64)
Patients included	4425 (±19,404)	62,625 (±27,431)	917.6 (±1633.4)	291.8 (±317.6)	721 (±625)	15,168 (±123,161)
Age (mean in years, number of studies)	54.2 (±6.4), *n* = 35	54.2 (±7.7), *n* = 18	53.2 (±5.5), *n* = 16	53.5 (±4.7), *n* = 11	52.9 (±4.2), *n* = 8	53.8 (±6.0), *n* = 88
Female (% of total patients, number of studies)	43.0%, *n* = 36	45.7%, *n* = 18	40.4%, *n* = 16	44.2%, *n* = 11	43.5%, *n* = 8	43.3%, *n* = 89
White race (% of total patients, number of studies)	72.7% (±12.6), *n* = 35	71.8% (±20.6), *n* = 18	69.9% (±16.6), *n* = 17	74.2% (±12.0), *n* = 11	72.4% (±11.5), *n* = 8	72.1% (±14.9), *n* = 89
Black race (% of total patients, number of studies)	19.3% (±10.8), *n* = 19	21.6% (±17.8), *n* = 12	18.3% (±11.6), *n* = 13	17.5% (±11.2), *n* = 7	13.2% (±12.1), *n* = 2	19.1% (±12.6). *n* = 53
Hispanic race (% of total patients, number of studies)	9.5% (±8.7), *n* = 10	11.9% (±10.4), *n* = 6	13.0% (±10.6), *n* = 10	7.3% (±), *n* = 1	9.0% (±7.2), *n* = 2	11.1% (±9.2), *n* = 29
Asian race (% of total patients, number of studies)	4.0% (±3.5), *n* = 6	1.5% (±1.6), *n* = 3	8.0% (±8.7), *n* = 9	2.7% (±0.4), *n* = 2	1.0% (±), *n* = 1	5.1% (±6.5), *n* = 21
Native American race (% of total patients, number of studies)	2.0% (±2.0), *n* = 4	1.8% (±), *n* = 1	1.4% (±1.2), *n* = 2	9.0% (±), *n* = 1	NA, *n* = 0	2.7% (±3.0), *n* = 8
Multiracial race (% of total patients, number of studies)	1.2% (±), *n* = 1	NA, *n* = 0	12.8% (±15.8), *n* = 2	NA, *n* = 0	NA, *n* = 0	8.9% (±13.0), *n* = 3
Other race (% of total patients, number of studies)	9.4% (±8.4), *n* = 17	3.8% (±3.5), *n* = 9	5.8% (±3.5), *n* = 7	5.0% (±2.2), *n* = 8	0.5% (±), *n* = 1	6.6% (±6.2), *n* = 42

**Table 2:** Differences between studies that reported only White vs. reported multiple races.

	Studies that reported White patients only (*N* = 35)	Studies that reported multiple races (*n* = 55)	*p* value
Journal			
AJRCC	4 (23.5%)	13 (76.5%)	
CC	3 (27.3%)	8 (72.7%)	0.116
CCM	16 (44.4%)	20 (55.6%)	
Chest	6 (33.3%)	12 (66.7%)	
ICM	6 (75%)	2 (25%)	
Study type			
Prospective cohort	20 (47.6%)	22 (52.4%)	
Retrospective cohort	5 (26.5%)	14 (73.7%)	0.241
Randomized-controlled trial	10 (34.5%)	19 (65.5%)	
Year of publication			
2011	0	8 (100%)	
2012	3 (50%)	3 (50%)	
2013	2 (40%)	3 (60%)	
2014	4 (44.4%)	5 (55.6%)	
2015	6 (40%)	9 (60%)	0.417
2016	8 (61.5%)	5 (38.5%)	
2017	6 (40%)	9 (60%)	
2018	3 (42.9%)	4 (57.1%)	
2019	1 (16.7%)	5 (83.3%)	
2020	2 (40%)	3 (60%)	
2021	0	1 (100%)	
Citations (median [SD])	46.6 (21.4)	57.7 (80.1)	0.426

## Data Availability

Data are available upon reasonable request to the corresponding author.

## Supporting Information

Studies that met the inclusion criteria and were analyzed in the manuscript.
