# Supplementary material for: ARDS Studies in Critical Care Journals: How Representative Are the Patients Studied?
Source: Crit Care Res Pract. 2025 Sep 10;2025:4060643. doi: 10.1155/ccrp/4060643 (PMC12443510; doi:10.1155/ccrp/4060643)
Supplement: Supporting Information — Additional supporting information can be found online in the Supporting Information section. [file 4060643.f1.docx]

| Study Title | First Author | Year | Number of Patients |
| --- | --- | --- | --- |
| A simple classification model for hospital mortality in patients with acute lung injury managed with lung protective ventilation | Brown et al (1) | 2011 | 2022 |
| Persistence of Community-Acquired Respiratory Distress Syndrome Toxin-Producing Mycoplasma pneumoniae in Refractory Asthma | Peters et al (2) | 2011 | 64 |
| Use of risk reclassification with multiple biomarkers improves mortality prediction in acute lung injury | Calfee et al (3) | 2011 | 1047 |
| Red blood cell transfusion and outcomes in patients with acute lung injury, sepsis and shock | Parsons et al (4) | 2011 | 285 |
| Short-term mortality prediction for acute lung injury patients: External validation of the Acute Respiratory Distress Syndrome Network prediction model | Damluji et al (5) | 2011 | 508 |
| Utilization patterns and patient outcomes associated with use of rescue therapies in acute lung injury | Walkey et al (6) | 2011 | 2632 |
| Randomized, Placebo-controlled Clinical Trial of an Aerosolized beta(2)-Agonist for Treatment of Acute Lung Injury | Matthay et al (7) | 2011 | 282 |
| Active and Passive Cigarette Smoking and Acute Lung Injury after Severe Blunt Trauma | Calfee et al (8) | 2011 | 144 |
| Macrolide Antibiotics and Survival in Patients With Acute Lung Injury | Walkey et al (9) | 2012 | 235 |
| Statin therapy as prevention against development of acute respiratory distress syndrome: An observational study | Bajwa et al (10) | 2012 | 2743 |
| Body mass index and acute kidney injury in the acute respiratory distress syndrome | Soto et al (11) | 2012 | 751 |
| The Adult Respiratory Distress Syndrome Cognitive Outcomes Study Long-Term Neuropsychological Function in Survivors of Acute Lung Injury | Mikkelsen et al (12) | 2012 | 731 |
| Association between inhaled nitric oxide treatment and long-term pulmonary function in survivors of acute respiratory distress syndrome | Dellinger et al (13) | 2012 | 92 |
| Inflammasome-regulated Cytokines Are Critical Mediators of Acute Lung Injury | Dolinay et al (14) | 2012 | 225 |
| Low plasma citrulline levels are associated with acute respiratory distress syndrome in patients with severe sepsis | Ware et al (15) | 2013 | 135 |
| Role of Diabetes in the Development of Acute Respiratory Distress Syndrome | Yu et al (16) | 2013 | 3827 |
| Growth differentiation factor-15 and prognosis in acute respiratory distress syndrome: a retrospective cohort study | Clark et al (17) | 2013 | 400 |
| Alcohol Screening Scores and 90-Day Outcomes in Patients With Acute Lung Injury | Clark et al (18) | 2013 | 1037 |
| Prognostic and Diagnostic Value of Plasma Soluble Suppression of Tumorigenicity-2 Concentrations in Acute Respiratory Distress Syndrome | Bajwa et al (19) | 2013 | 826 |
| Screening for critical illness polyneuromyopathy with single nerve conduction studies | Moss et al (20) | 2014 | 64 |
| Detection of Fibroproliferation by Chest High-Resolution CT Scan in Resolving ARDS | Burnham et al (21) | 2014 | 82 |
| Statins and Delirium During Critical Illness: A Multicenter, Prospective Cohort Study | Morandi et al (22) | 2014 | 763 |
| The Role of Potentially Preventable Hospital Exposures in the Development of Acute Respiratory Distress Syndrome: A Population-Based Study | Ahmed et al (23) | 2014 | 828 |
| Characteristics and Outcomes of Patients Hospitalized Following Pulmonary Aspiration | Lee et al (24) | 2014 | 5584 |
| Treatment With Neuromuscular Blocking Agents and the Risk of In-Hospital Mortality Among Mechanically Ventilated Patients With Severe Sepsis | Steingrub et al (25) | 2014 | 7864 |
| Prevalence and Impact of Active and Passive Cigarette Smoking in Acute Respiratory Distress Syndrome | Hsieh et al (26) | 2014 | 381 |
| Red Blood Cells Induce Necroptosis of Lung Endothelial Cells and Increase Susceptibility to Lung Inflammation | Qing et al (27) | 2014 | 37 |
| Long-Term Survival in Patients With Severe Acute Respiratory Distress Syndrome and Rescue Therapies for Refractory Hypoxemia | Khandelwal et al (28) | 2014 | 428 |
| Development and Validation of a Mortality Prediction Model for Patients Receiving 14 Days of Mechanical Ventilation | Hough et al (29) | 2015 | 736 |
| Six-month quality-of-life and functional status of acute respiratory distress syndrome survivors compared to patients at risk: a population-based study | Biehl et al (30) | 2015 | 67 |
| Long-Term Ozone Exposure Increases the Risk of Developing the Acute Respiratory Distress Syndrome | Ware et al (31) | 2015 | 1558 |
| Cigarette Smoke Exposure and the Acute Respiratory Distress Syndrome | Calfee et al (32) | 2015 | 426 |
| Clinical Characteristics and Outcomes Are Similar in ARDS Diagnosed by Oxygen Saturation/FIO2 Ratio Compared With PaO2/FIO2 Ratio | Chen et al (33) | 2015 | 362 |
| Prehospital Aspirin Use Is Associated With Reduced Risk of Acute Respiratory Distress Syndrome in Critically Ill Patients: A Propensity-Adjusted Analysis | Chen et al (34) | 2015 | 1149 |
| A Randomized Dose-Escalation Study of the Safety and Anti-Inflammatory Activity of the p38 Mitogen-Activated Protein Kinase Inhibitor Dilmapimod in Severe Trauma Subjects at Risk for Acute Respiratory Distress Syndrome | Christie et al (35) | 2015 | 77 |
| Plasma soluble thrombomodulin levels are associated with mortality in the acute respiratory distress syndrome | Sapru et al (36) | 2015 | 994 |
| Construct Validity and Minimal Important Difference of 6-Minute Walk Distance in Survivors of Acute Respiratory Failure | Chan et al (37) | 2015 | 651 |
| Protocols and Hospital Mortality in Critically Ill Patients: The United States Critical Illness and Injury Trials Group Critical Illness Outcomes Study | Sevransky et al (38) | 2015 | 6179 |
| Platelet Count Mediates the Contribution of a Genetic Variant in LRRC16A to ARDS Risk | Wei et al (39) | 2015 | 1655 |
| Mechanical Ventilation and ARDS in the ED A Multicenter, Observational, Prospective, Cross-sectional Study | Fuller et al (40) | 2015 | 219 |
| Distinct Molecular Phenotypes of Direct vs Indirect ARDS in Single-Center and Multicenter Studies | Calfee et al (41) | 2015 | 953 |
| Quality of Communication in the ICU and Surrogate's Understanding of Prognosis | Chiarchiaro et al (42) | 2015 | 251 |
| The Association between Acute Respiratory Distress Syndrome, Delirium, and In-Hospital Mortality in Intensive Care Unit Patients | Hsieh et al (43) | 2015 | 532 |
| Low Plasma Levels of Adiponectin Do Not Explain Acute Respiratory Distress Syndrome Risk: a Prospective Cohort Study of Patients with Severe Sepsis | Palakshappa et al (44) | 2016 | 163 |
| Impact of Initial Central Venous Pressure on Outcomes of Conservative Versus Liberal Fluid Management in Acute Respiratory Distress Syndrome | Semler et al (45) | 2016 | 609 |
| Plasma Concentrations of Soluble Suppression of Tumorigenicity-2 and Interleukin-6 Are Predictive of Successful Liberation From Mechanical Ventilation in Patients With the Acute Respiratory Distress Syndrome | Alladina et al (46) | 2016 | 1581 |
| B-Type Natriuretic Peptide, Aldosterone, and Fluid Management in ARDS | Semler et al (47) | 2016 | 1258 |
| Psychiatric Symptoms in Acute Respiratory Distress Syndrome Survivors: A 1-Year National Multicenter Study | Huang et al (48) | 2016 | 613 |
| Mortality and pulmonary mechanics in relation to respiratory system and transpulmonary driving pressures in ARDS | Kassis et al (49) | 2016 | 56 |
| Clinical Predictors of Hospital Mortality Differ Between Direct and Indirect ARDS | Luo et al (50) | 2016 | 834 |
| Interleukin-17A Is Associated With Alveolar Inflammation and Poor Outcomes in Acute Respiratory Distress Syndrome | Mikacenic et al (51) | 2016 | 226 |
| Evaluating Physical Outcomes in Acute Respiratory Distress Syndrome Survivors: Validity, Responsiveness, and Minimal Important Difference of 4-Meter Gait Speed Test | Chan et al (52) | 2016 | 306 |
| Reciprocal Risk of Acute Kidney Injury and Acute Respiratory Distress Syndrome in Critically III Burn Patients | Clemens et al (53) | 2016 | 830 |
| Neutropenic sepsis is associated with distinct clinical and biological characteristics: a cohort study of severe sepsis | Reilly et al (54) | 2016 | 794 |
| Nonlinear Imputation of PaO2/FIO2 From SpO(2)/FIO2 Among Patients With Acute Respiratory Distress Syndrome | Brown et al (55) | 2016 | 1184 |
| Lung Injury Prediction Score in Hospitalized Patients at Risk of Acute Respiratory Distress Syndrome | Soto et al (56) | 2016 | 900 |
| External validation of a biomarker and clinical prediction model for hospital mortality in acute respiratory distress syndrome | Zhao et al (57) | 2017 | 1922 |
| Healthcare Resource Use and Costs in Long-Term Survivors of Acute Respiratory Distress Syndrome: A 5-Year Longitudinal Cohort Study | Ruhl et al (58) | 2017 | 138 |
| Randomized Clinical Trial of a Combination of an Inhaled Corticosteroid and Beta Agonist in Patients at Risk of Developing the Acute Respiratory Distress Syndrome | Festic et al (59) | 2017 | 60 |
| Healthcare utilization and costs in ARDS survivors: a 1-year longitudinal national US multicenter study | Ruhl et al (60) | 2017 | 859 |
| Physicians Rarely Elicit Critically Ill Patients' Previously Expressed Treatment Preferences in Intensive Care Units | Chiarchiaro et al (61) | 2017 | 245 |
| Joblessness and Lost Earnings after ARDS in a 1-Year National Multicenter Study Abstracts | Kamdar et al (62) | 2017 | 922 |
| Late-onset moderate to severe acute respiratory distress syndrome is associated with shorter survival and higher mortality: a two-stage association study | Zhang et al (63) | 2017 | 876 |
| A Two Biomarker Model Predicts Mortality in the Critically Ill with Sepsis | Mikacenic et al (64) | 2017 | 1925 |
| A Missense Genetic Variant in LRRC16A/CARMIL1 Improves Acute Respiratory Distress Syndrome Survival by Attenuating Platelet Count Decline | Wei et al (65) | 2017 | 414 |
| Pilot Feasibility Study of Therapeutic Hypothermia for Moderate to Severe Acute Respiratory Distress Syndrome | Slack et al (66) | 2017 | 58 |
| Oxygenation Saturation Index Predicts Clinical Outcomes in ARDS | DesPrez et al (67) | 2017 | 329 |
| Prevalence and Prognostic Association of Circulating Troponin in the Acute Respiratory Distress Syndrome | Metkus et al (68) | 2017 | 1057 |
| Hyaluronic acid is associated with organ dysfunction in acute respiratory distress syndrome | Esposito et al (69) | 2017 | 86 |
| A Quasi-Experimental, Before-After Trial Examining the Impact of an Emergency Department Mechanical Ventilator Protocol on Clinical Outcomes and Lung-Protective Ventilation in Acute Respiratory Distress Syndrome | Fuller et al (70) | 2017 | 229 |
| Lung Microbiota Is Related to Smoking Status and to Development of Acute Respiratory Distress Syndrome in Critically Ill Trauma Patients | Panzer et al (71) | 2017 | 74 |
| The Association Between Ventilator Dyssynchrony, Delivered Tidal Volume, and Sedation Using a Novel Automated Ventilator Dyssynchrony Detection Algorithm | Sottile et al (72) | 2018 | 62 |
| Psychiatric symptoms after acute respiratory distress syndrome: a 5-year longitudinal study | Bienvenu et al (73) | 2018 | 186 |
| The Association Between Acute Respiratory Distress Syndrome Hospital Case Volume and Mortality in a US Cohort, 2002-2011 | Ike et al (74) | 2018 | 117204 |
| An Observational Study of the Efficacy of Cisatracurium Compared with Vecuronium in Patients with or at Risk for Acute Respiratory Distress Syndrome | Sottile et al (75) | 2018 | 6925 |
| Peripheral and Alveolar Cell Transcriptional Programs Are Distinct in Acute Respiratory Distress Syndrome | Morrell et al (76) | 2018 | 26 |
| Neutrophil extracellular traps (NETs) are increased in the alveolar spaces of patients with ventilator-associated pneumonia | Mikacenic et al (77) | 2018 | 100 |
| Early Intravascular Events Are Associated with Development of Acute Respiratory Distress Syndrome A Substudy of the LIPS-A Clinical Trial | Abdulnour et al (78) | 2018 | 367 |
| Association of Elevated Plasma Interleukin 18 level with Increased Mortality in a Clinical Trial of Statin Treatment for ARDS | Rogers et al (79) | 2019 | 683 |
| Screening for posttraumatic stress disorder in ARDS survivors: validation of the Impact of Event Scale-6 (IES-6) | Hosey et al (80) | 2019 | 1001 |
| Rapidly Improving ARDS in Therapeutic Randomized Controlled Trials | Schenck et al (81) | 2019 | 1909 |
| Impact of Long-Term Exposures to Ambient PM2.5 and Ozone on ARDS Risk for Older Adults in the United States | Rhee et al (82) | 2019 | 1164784 |
| Physiologic Analysis and Clinical Performance of the Ventilatory Ratio in Acute Respiratory Distress Syndrome | Sinha et al (83) | 2019 | 520 |
| Development of a biomarker mortality risk model in acute respiratory distress syndrome | Bime et al (84) | 2019 | 252 |
| Acute respiratory distress syndrome-attributable mortality in critically ill patients with sepsis | Auriemma et al (85) | 2020 | 811 |
| Fatigue Symptoms During the First Year Following ARDS | Neufeld et al (86) | 2020 | 732 |
| Plasma sRAGE Acts as a Genetically Regulated Causal Intermediate in Sepsis-associated Acute Respiratory Distress Syndrome | Jones et al (87) | 2020 | 672 |
| A lung rescue team improves survival in obesity with acute respiratory distress syndrome | Florio et al (88) | 2020 | 120 |
| Plasma Mitochondrial DNA Levels Are Associated With ARDS in Trauma and Sepsis Patients | Faust et al (89) | 2020 | 344 |
| Ventilatory Mechanics in Early vs Late Intubation in a Cohort of Coronavirus Disease 2019 Patients With ARDS A Single Center's Experience | Pandya et al (90) | 2021 | 75 |

Supplementary table 1 Studies that met the inclusion criteria

**References**

1. Brown LM, Calfee CS, Matthay MA, Brower RG, Thompson BT, Checkley W, National Institutes of Health Acute Respiratory Distress Syndrome Network I. A simple classification model for hospital mortality in patients with acute lung injury managed with lung protective ventilation. Crit Care Med. 2011;39(12):2645-51.

2. Peters J, Singh H, Brooks EG, Diaz J, Kannan TR, Coalson JJ, et al. Persistence of community-acquired respiratory distress syndrome toxin-producing Mycoplasma pneumoniae in refractory asthma. Chest. 2011;140(2):401-7.

3. Calfee CS, Ware LB, Glidden DV, Eisner MD, Parsons PE, Thompson BT, et al. Use of risk reclassification with multiple biomarkers improves mortality prediction in acute lung injury. Crit Care Med. 2011;39(4):711-7.

4. Parsons EC, Hough CL, Seymour CW, Cooke CR, Rubenfeld GD, Watkins TR, Network NA. Red blood cell transfusion and outcomes in patients with acute lung injury, sepsis and shock. Crit Care. 2011;15(5):R221.

5. Damluji A, Colantuoni E, Mendez-Tellez PA, Sevransky JE, Fan E, Shanholtz C, et al. Short-term mortality prediction for acute lung injury patients: external validation of the Acute Respiratory Distress Syndrome Network prediction model. Crit Care Med. 2011;39(5):1023-8.

6. Walkey AJ, Wiener RS. Utilization patterns and patient outcomes associated with use of rescue therapies in acute lung injury. Crit Care Med. 2011;39(6):1322-8.

7. National Heart L, Blood Institute Acute Respiratory Distress Syndrome Clinical Trials N, Matthay MA, Brower RG, Carson S, Douglas IS, et al. Randomized, placebo-controlled clinical trial of an aerosolized β₂-agonist for treatment of acute lung injury. Am J Respir Crit Care Med. 2011;184(5):561-8.

8. Calfee CS, Matthay MA, Eisner MD, Benowitz N, Call M, Pittet J-F, Cohen MJ. Active and passive cigarette smoking and acute lung injury after severe blunt trauma. Am J Respir Crit Care Med. 2011;183(12):1660-5.

9. Walkey AJ, Wiener RS. Macrolide antibiotics and survival in patients with acute lung injury. Chest. 2012;141(5):1153-9.

10. Bajwa EK, Malhotra CK, Thompson BT, Christiani DC, Gong MN. Statin therapy as prevention against development of acute respiratory distress syndrome: an observational study. Crit Care Med. 2012;40(5):1470-7.

11. Soto GJ, Frank AJ, Christiani DC, Gong MN. Body mass index and acute kidney injury in the acute respiratory distress syndrome. Crit Care Med. 2012;40(9):2601-8.

12. Mikkelsen ME, Christie JD, Lanken PN, Biester RC, Thompson BT, Bellamy SL, et al. The adult respiratory distress syndrome cognitive outcomes study: long-term neuropsychological function in survivors of acute lung injury. Am J Respir Crit Care Med. 2012;185(12):1307-15.

13. Dellinger RP, Trzeciak SW, Criner GJ, Zimmerman JL, Taylor RW, Usansky H, et al. Association between inhaled nitric oxide treatment and long-term pulmonary function in survivors of acute respiratory distress syndrome. Crit Care. 2012;16(2):R36.

14. Dolinay T, Kim YS, Howrylak J, Hunninghake GM, An CH, Fredenburgh L, et al. Inflammasome-regulated cytokines are critical mediators of acute lung injury. Am J Respir Crit Care Med. 2012;185(11):1225-34.

15. Ware LB, Magarik JA, Wickersham N, Cunningham G, Rice TW, Christman BW, et al. Low plasma citrulline levels are associated with acute respiratory distress syndrome in patients with severe sepsis. Crit Care. 2013;17(1):R10.

16. Yu S, Christiani DC, Thompson BT, Bajwa EK, Gong MN. Role of diabetes in the development of acute respiratory distress syndrome. Crit Care Med. 2013;41(12):2720-32.

17. Clark BJ, Bull TM, Benson AB, Stream AR, Macht M, Gaydos J, et al. Growth differentiation factor-15 and prognosis in acute respiratory distress syndrome: a retrospective cohort study. Crit Care. 2013;17(3):R92.

18. Clark BJ, Williams A, Feemster LMC, Bradley KA, Macht M, Moss M, et al. Alcohol screening scores and 90-day outcomes in patients with acute lung injury. Crit Care Med. 2013;41(6):1518-25.

19. Bajwa EK, Volk JA, Christiani DC, Harris RS, Matthay MA, Thompson BT, et al. Prognostic and diagnostic value of plasma soluble suppression of tumorigenicity-2 concentrations in acute respiratory distress syndrome. Crit Care Med. 2013;41(11):2521-31.

20. Moss M, Yang M, Macht M, Sottile P, Gray L, McNulty M, Quan D. Screening for critical illness polyneuromyopathy with single nerve conduction studies. Intensive Care Med. 2014;40(5):683-90.

21. Burnham EL, Hyzy RC, Paine R, 3rd, Kelly AM, Quint LE, Lynch D, et al. Detection of fibroproliferation by chest high-resolution CT scan in resolving ARDS. Chest. 2014;146(5):1196-204.

22. Morandi A, Hughes CG, Thompson JL, Pandharipande PP, Shintani AK, Vasilevskis EE, et al. Statins and delirium during critical illness. Crit Care Med. 2014;42(8):1899-909.

23. Ahmed AH, Litell JM, Malinchoc M, Kashyap R, Schiller HJ, Pannu SR, et al. The role of potentially preventable hospital exposures in the development of acute respiratory distress syndrome: a population-based study. Crit Care Med. 2014;42(1):31-9.

24. Lee A, Festic E, Park PK, Raghavendran K, Dabbagh O, Adesanya A, et al. Characteristics and outcomes of patients hospitalized following pulmonary aspiration. Chest. 2014;146(4):899-907.

25. Steingrub JS, Lagu T, Rothberg MB, Nathanson BH, Raghunathan K, Lindenauer PK. Treatment with neuromuscular blocking agents and the risk of in-hospital mortality among mechanically ventilated patients with severe sepsis. Crit Care Med. 2014;42(1):90-6.

26. Hsieh SJ, Zhuo H, Benowitz NL, Thompson BT, Liu KD, Matthay MA, Calfee CS. Prevalence and impact of active and passive cigarette smoking in acute respiratory distress syndrome. Crit Care Med. 2014;42(9):2058-68.

27. Qing DY, Conegliano D, Shashaty MG, Seo J, Reilly JP, Worthen GS, et al. Red blood cells induce necroptosis of lung endothelial cells and increase susceptibility to lung inflammation. Am J Respir Crit Care Med. 2014;190(11):1243-54.

28. Khandelwal N, Hough CL, Bansal A, Veenstra DL, Treggiari MM. Long-term survival in patients with severe acute respiratory distress syndrome and rescue therapies for refractory hypoxemia. Crit Care Med. 2014;42(7):1610-8.

29. Hough CL, Caldwell ES, Cox CE, Douglas IS, Kahn JM, White DB, et al. Development and validation of a mortality prediction model for patients receiving 14 days of mechanical ventilation. Crit Care Med. 2015;43(11):2339-45.

30. Biehl M, Kashyap R, Ahmed AH, Reriani MK, Ofoma UR, Wilson GA, et al. Six-month quality-of-life and functional status of acute respiratory distress syndrome survivors compared to patients at risk: a population-based study. Crit Care. 2015;19(1):356.

31. Ware LB, Zhao Z, Koyama T, May AK, Matthay MA, Lurmann FW, et al. Long-term ozone exposure increases the risk of developing the acute respiratory distress syndrome. Am J Respir Crit Care Med. 2016;193(10):1143-50.

32. Calfee CS, Matthay MA, Kangelaris KN, Siew ED, Janz DR, Bernard GR, et al. Cigarette smoke exposure and the acute respiratory distress syndrome. Crit Care Med. 2015;43(9):1790-7.

33. Chen W, Janz DR, Shaver CM, Bernard GR, Bastarache JA, Ware LB. Clinical characteristics and outcomes are similar in ARDS diagnosed by oxygen saturation/Fio2 ratio compared with Pao2/Fio2 ratio. Chest. 2015;148(6):1477-83.

34. Chen W, Janz DR, Bastarache JA, May AK, O'Neal HR, Jr., Bernard GR, Ware LB. Prehospital aspirin use is associated with reduced risk of acute respiratory distress syndrome in critically ill patients: a propensity-adjusted analysis. Crit Care Med. 2015;43(4):801-7.

35. Christie JD, Vaslef S, Chang PK, May AK, Gunn SR, Yang S, et al. A randomized dose-escalation study of the safety and anti-inflammatory activity of the p38 mitogen-activated protein kinase inhibitor dilmapimod in severe trauma subjects at risk for acute respiratory distress syndrome. Crit Care Med. 2015;43(9):1859-69.

36. Sapru A, Calfee CS, Liu KD, Kangelaris K, Hansen H, Pawlikowska L, et al. Plasma soluble thrombomodulin levels are associated with mortality in the acute respiratory distress syndrome. Intensive Care Med. 2015;41(3):470-8.

37. Chan KS, Pfoh ER, Denehy L, Elliott D, Holland AE, Dinglas VD, Needham DM. Construct validity and minimal important difference of 6-minute walk distance in survivors of acute respiratory failure. Chest. 2015;147(5):1316-26.

38. Sevransky JE, Checkley W, Herrera P, Pickering BW, Barr J, Brown SM, et al. Protocols and hospital mortality in critically ill patients: The United States Critical Illness and Injury Trials Group Critical Illness Outcomes Study. Crit Care Med. 2015;43(10):2076-84.

39. Wei Y, Wang Z, Su L, Chen F, Tejera P, Bajwa EK, et al. Platelet count mediates the contribution of a genetic variant in LRRC16A to ARDS risk. Chest. 2015;147(3):607-17.

40. Fuller BM, Mohr NM, Miller CN, Deitchman AR, Levine BJ, Castagno N, et al. Mechanical ventilation and ARDS in the ED: A multicenter, observational, prospective, cross-sectional study. Chest. 2015;148(2):365-74.

41. Calfee CS, Janz DR, Bernard GR, May AK, Kangelaris KN, Matthay MA, Ware LB. Distinct molecular phenotypes of direct vs indirect ARDS in single-center and multicenter studies. Chest. 2015;147(6):1539-48.

42. Chiarchiaro J, Buddadhumaruk P, Arnold RM, White DB. Quality of communication in the ICU and surrogate's understanding of prognosis. Crit Care Med. 2015;43(3):542-8.

43. Hsieh SJ, Soto GJ, Hope AA, Ponea A, Gong MN. The association between acute respiratory distress syndrome, delirium, and in-hospital mortality in intensive care unit patients. Am J Respir Crit Care Med. 2015;191(1):71-8.

44. Palakshappa JA, Anderson BJ, Reilly JP, Shashaty MGS, Ueno R, Wu Q, et al. Low plasma levels of adiponectin do not explain acute respiratory distress syndrome risk: A prospective cohort study of patients with severe sepsis. Crit Care. 2016;20(1).

45. Semler MW, Wheeler AP, Thompson BT, Bernard GR, Wiedemann HP, Rice TW, et al. Impact of initial central venous pressure on outcomes of conservative versus liberal fluid management in acute respiratory distress syndrome. Crit Care Med. 2016;44(4):782-9.

46. Alladina JW, Levy SD, Hibbert KA, Januzzi JL, Harris RS, Matthay MA, et al. Plasma concentrations of soluble suppression of tumorigenicity-2 and interleukin-6 are predictive of successful liberation from mechanical ventilation in patients with the acute respiratory distress syndrome. Crit Care Med. 2016;44(9):1735-43.

47. Semler MW, Marney AM, Rice TW, Nian H, Yu C, Wheeler AP, Brown NJ. B-Type Natriuretic Peptide, Aldosterone, and Fluid Management in ARDS. Chest. 2016;150(1):102-11.

48. Huang M, Parker AM, Bienvenu OJ, Dinglas VD, Colantuoni E, Hopkins RO, et al. Psychiatric symptoms in acute respiratory distress syndrome survivors: A 1-year national multicenter study. Crit Care Med. 2016;44(5):954-65.

49. Baedorf Kassis E, Loring SH, Talmor D. Mortality and pulmonary mechanics in relation to respiratory system and transpulmonary driving pressures in ARDS. Intensive Care Med. 2016;42(8):1206-13.

50. Luo L, Shaver CM, Zhao Z, Koyama T, Calfee CS, Bastarache JA, Ware LB. Clinical predictors of hospital mortality differ between direct and indirect ARDS. Chest. 2017;151(4):755-63.

51. Mikacenic C, Hansen EE, Radella F, Gharib SA, Stapleton RD, Wurfel MM. Interleukin-17A is associated with alveolar inflammation and poor outcomes in acute respiratory distress syndrome. Crit Care Med. 2016;44(3):496-502.

52. Chan KS, Aronson Friedman L, Dinglas VD, Hough CL, Morris PE, Mendez-Tellez PA, et al. Evaluating physical outcomes in acute respiratory distress syndrome survivors. Crit Care Med. 2016;44(5):859-68.

53. Clemens MS, Stewart IJ, Sosnov JA, Howard JT, Belenkiy SM, Sine CR, et al. Reciprocal risk of acute kidney injury and acute respiratory distress syndrome in critically ill burn patients. Crit Care Med. 2016;44(10):e915-e22.

54. Reilly JP, Anderson BJ, Hudock KM, Dunn TG, Kazi A, Tommasini A, et al. Neutropenic sepsis is associated with distinct clinical and biological characteristics: a cohort study of severe sepsis. Crit Care. 2016;20(1).

55. Brown SM, Grissom CK, Moss M, Rice TW, Schoenfeld D, Hou PC, et al. Nonlinear Imputation of Pa o 2 /F io 2 From Sp o 2 /F io 2 Among Patients With Acute Respiratory Distress Syndrome. Chest. 2016;150(2):307-13.

56. Soto GJ, Kor DJ, Park PK, Hou PC, Kaufman DA, Kim M, et al. Lung Injury Prediction Score in hospitalized patients at risk of acute respiratory distress syndrome. Crit Care Med. 2016;44(12):2182-91.

57. Zhao Z, Wickersham N, Kangelaris KN, May AK, Bernard GR, Matthay MA, et al. External validation of a biomarker and clinical prediction model for hospital mortality in acute respiratory distress syndrome. Intensive Care Med. 2017;43(8):1123-31.

58. Ruhl AP, Huang M, Colantuoni E, Lord RK, Dinglas VD, Chong A, et al. Healthcare resource use and costs in long-term survivors of acute respiratory distress syndrome: A 5-year longitudinal cohort study. Crit Care Med. 2017;45(2):196-204.

59. Festic E, Carr GE, Cartin-Ceba R, Hinds RF, Banner-Goodspeed V, Bansal V, et al. Randomized clinical trial of a combination of an inhaled corticosteroid and beta agonist in patients at risk of developing the acute respiratory distress syndrome. Crit Care Med. 2017;45(5):798-805.

60. Ruhl AP, With the National Institutes of Health NHL, Blood Institute Acute Respiratory Distress Syndrome N, Huang M, Colantuoni E, Karmarkar T, et al. Healthcare utilization and costs in ARDS survivors: a 1-year longitudinal national US multicenter study. Intensive Care Med. 2017;43(7):980-91.

61. Chiarchiaro J, Ernecoff NC, Scheunemann LP, Hough CL, Carson SS, Peterson MW, et al. Physicians rarely elicit critically ill patients' previously expressed treatment preferences in intensive care units. Am J Respir Crit Care Med. 2017;196(2):242-5.

62. Kamdar BB, Huang M, Dinglas VD, Colantuoni E, von Wachter TM, Hopkins RO, et al. Joblessness and lost earnings after acute respiratory distress syndrome in a 1-year national multicenter study. Am J Respir Crit Care Med. 2017;196(8):1012-20.

63. Zhang R, Wang Z, Tejera P, Frank AJ, Wei Y, Su L, et al. Late-onset moderate to severe acute respiratory distress syndrome is associated with shorter survival and higher mortality: a two-stage association study. Intensive Care Med. 2017;43(3):399-407.

64. Mikacenic C, Price BL, Harju-Baker S, O'Mahony DS, Robinson-Cohen C, Radella F, et al. A two-biomarker model predicts mortality in the critically ill with sepsis. Am J Respir Crit Care Med. 2017;196(8):1004-11.

65. Wei Y, Tejera P, Wang Z, Zhang R, Chen F, Su L, et al. A Missense Genetic Variant in LRRC16A/CARMIL1 Improves Acute Respiratory Distress Syndrome Survival by Attenuating Platelet Count Decline. Am J Respir Crit Care Med. 2017;195(10):1353-61.

66. Slack DF, Corwin DS, Shah NG, Shanholtz CB, Verceles AC, Netzer G, et al. Pilot feasibility study of therapeutic hypothermia for moderate to severe acute respiratory distress syndrome. Crit Care Med. 2017;45(7):1152-9.

67. DesPrez K, McNeil JB, Wang C, Bastarache JA, Shaver CM, Ware LB. Oxygenation saturation index predicts clinical outcomes in ARDS. Chest. 2017;152(6):1151-8.

68. Metkus TS, Guallar E, Sokoll L, Morrow D, Tomaselli G, Brower R, et al. Prevalence and prognostic association of circulating troponin in the acute respiratory distress syndrome. Crit Care Med. 2017;45(10):1709-17.

69. Esposito AJ, Bhatraju PK, Stapleton RD, Wurfel MM, Mikacenic C. Hyaluronic acid is associated with organ dysfunction in acute respiratory distress syndrome. Crit Care. 2017;21(1).

70. Fuller BM, Ferguson IT, Mohr NM, Drewry AM, Palmer C, Wessman BT, et al. A quasi-experimental, before-after trial examining the impact of an emergency department mechanical ventilator protocol on clinical outcomes and lung-protective ventilation in acute respiratory distress syndrome. Crit Care Med. 2017;45(4):645-52.

71. Panzer AR, Lynch SV, Langelier C, Christie JD, McCauley K, Nelson M, et al. Lung Microbiota is related to smoking status and to development of acute respiratory distress syndrome in critically ill trauma patients. Am J Respir Crit Care Med. 2018;197(5):621-31.

72. Sottile PD, Albers D, Higgins C, McKeehan J, Moss MM. The association between ventilator dyssynchrony, delivered tidal volume, and sedation using a novel automated ventilator dyssynchrony detection algorithm. Crit Care Med. 2018;46(2):e151-e7.

73. Bienvenu OJ, Friedman LA, Colantuoni E, Dinglas VD, Sepulveda KA, Mendez-Tellez P, et al. Psychiatric symptoms after acute respiratory distress syndrome: a 5-year longitudinal study. Intensive Care Med. 2018;44(1):38-47.

74. Ike JD, Kempker JA, Kramer MR, Martin GS. The association between acute respiratory distress syndrome hospital case volume and mortality in a U.s. cohort, 2002-2011. Crit Care Med. 2018;46(5):764-73.

75. Sottile PD, Kiser TH, Burnham EL, Ho PM, Allen RR, Vandivier RW, et al. An observational study of the efficacy of cisatracurium compared with vecuronium in patients with or at risk for acute respiratory distress syndrome. Am J Respir Crit Care Med. 2018;197(7):897-904.

76. Morrell ED, Radella F, 2nd, Manicone AM, Mikacenic C, Stapleton RD, Gharib SA, Wurfel MM. Peripheral and alveolar cell transcriptional programs are distinct in acute respiratory distress syndrome. Am J Respir Crit Care Med. 2018;197(4):528-32.

77. Mikacenic C, Moore R, Dmyterko V, West TE, Altemeier WA, Liles WC, Lood C. Neutrophil extracellular traps (NETs) are increased in the alveolar spaces of patients with ventilator-associated pneumonia. Crit Care. 2018;22(1):358.

78. Abdulnour R-EE, Gunderson T, Barkas I, Timmons JY, Barnig C, Gong M, et al. Early intravascular events are associated with development of acute respiratory distress syndrome. A substudy of the LIPS-A clinical trial. Am J Respir Crit Care Med. 2018;197(12):1575-85.

79. Rogers AJ, Guan J, Trtchounian A, Hunninghake GM, Kaimal R, Desai M, et al. Association of elevated plasma interleukin-18 level with increased mortality in a clinical trial of statin treatment for acute respiratory distress syndrome. Crit Care Med. 2019;47(8):1089-96.

80. Hosey MM, Leoutsakos JS, Li X, Dinglas VD, Bienvenu OJ, Parker AM, et al. Screening for posttraumatic stress disorder in ARDS survivors: validation of the Impact of Event Scale-6 (IES-6). Crit Care. 2019;23(1):276.

81. Schenck EJ, Oromendia C, Torres LK, Berlin DA, Choi AMK, Siempos II. Rapidly improving ARDS in therapeutic randomized controlled trials. Chest. 2019;155(3):474-82.

82. Rhee J, Dominici F, Zanobetti A, Schwartz J, Wang Y, Di Q, et al. Impact of long-term exposures to ambient PM2.5 and ozone on ARDS risk for older adults in the United States. Chest. 2019;156(1):71-9.

83. Sinha P, Calfee CS, Beitler JR, Soni N, Ho K, Matthay MA, Kallet RH. Physiologic analysis and clinical performance of the ventilatory ratio in acute respiratory distress syndrome. Am J Respir Crit Care Med. 2019;199(3):333-41.

84. Bime C, Casanova N, Oita RC, Ndukum J, Lynn H, Camp SM, et al. Development of a biomarker mortality risk model in acute respiratory distress syndrome. Crit Care. 2019;23(1):410.

85. Auriemma CL, Zhuo H, Delucchi K, Deiss T, Liu T, Jauregui A, et al. Acute respiratory distress syndrome-attributable mortality in critically ill patients with sepsis. Intensive Care Med. 2020;46(6):1222-31.

86. Neufeld KJ, Leoutsakos J-MS, Yan H, Lin S, Zabinski JS, Dinglas VD, et al. Fatigue symptoms during the first year following ARDS. Chest. 2020;158(3):999-1007.

87. Jones TK, Feng R, Kerchberger VE, Reilly JP, Anderson BJ, Shashaty MGS, et al. Plasma sRAGE acts as a genetically regulated causal intermediate in sepsis-associated acute respiratory distress syndrome. Am J Respir Crit Care Med. 2020;201(1):47-56.

88. Florio G, Ferrari M, Bittner EA, De Santis Santiago R, Pirrone M, Fumagalli J, et al. A lung rescue team improves survival in obesity with acute respiratory distress syndrome. Critical Care. 2020;24(1):4.

89. Faust HE, Reilly JP, Anderson BJ, Ittner CAG, Forker CM, Zhang P, et al. Plasma mitochondrial DNA levels are associated with ARDS in trauma and sepsis patients. Chest. 2020;157(1):67-76.

90. Pandya A, Kaur NA, Sacher D, O'Corragain O, Salerno D, Desai P, et al. Ventilatory mechanics in early vs late intubation in a cohort of Coronavirus disease 2019 patients with ARDS: A single center's experience. Chest. 2021;159(2):653-6.
